# Measurement of Sterigmatocystin Concentrations in Urine for Monitoring the Contamination of Cattle Feed

**DOI:** 10.3390/toxins6113117

**Published:** 2014-11-04

**Authors:** Yasuo Fushimi, Mitsuhiro Takagi, Seiichi Uno, Emiko Kokushi, Masayuki Nakamura, Hiroshi Hasunuma, Urara Shinya, Eisaburo Deguchi, Johanna Fink-Gremmels

**Affiliations:** 1 United Graduate School of Veterinary Science, Yamaguchi University, Yamaguchi 753-8515, Japan; E-Mails: yf19801113@hotmail.com (Y.F.); urara@nosai-soo.com (U.S.); deguchi@agri.kagoshima-u.ac.jp (E.D.); 2 Shepherd Central Livestock Clinic, Kagoshima 899-1611, Japan; E-Mail: hasu@fa3.so-net.ne.jp; 3 Joint Faculty of Veterinary Medicine, Kagoshima University, Kagoshima 890-0065, Japan; 4 Faculty of Fisheries, Kagoshima University, Kagoshima 890-0056, Japan; E-Mails: uno@fish.kagoshima-u.ac.jp (S.U.); kokushi@fish.kagoshima-u.ac.jp (E.K.); 5 Faculty of Agriculture, Kagoshima University, Kagoshima 890-0056, Japan; E-Mail: masa@agri.kagoshima-u.ac.jp; 6 Soo Agriculture Mutual Aid Association, Kagoshima 899-8212, Japan; 7 Faculty of Veterinary Medicine, Utrecht University, Yalelaan 104, The Netherlands; E-Mail: J.Fink@uu.nl

**Keywords:** cattle, sterigmatocystin, urine, LC-MS/MS

## Abstract

This study aimed (1) at determining the levels of the fungal toxin sterigmatocystin (STC) in the feed and urine of cattle and (2) at evaluating the effects of supplementing the feed with a mycotoxin adsorbent (MA) on STC concentrations in urine. Two herds of female Japanese Black cattle were used in this study. The cattle in each herd were fed a standard ration containing rice straw from different sources and a standard concentrate; two groups of cattle from each herd (*n* = six per group) received the commercial MA, mixed with the concentrate or given as top-dressing, whereas a third group received no supplement and served as control. Urine and feed samples were collected at various time points throughout the experiment. STC concentrations were measured using liquid chromatography-tandem mass spectrometry (LC-TMS). STC concentrations in straw were higher in Herd 1 (range 0.15–0.24 mg/kg DM) than in Herd 2 (range <0.01–0.06 mg/kg DM). In Herd 1, STC concentrations in urine significantly declined 2 weeks after replacing the contaminated feed, whereas MA supplementation had no effect. In conclusion, mycotoxins in urine samples are useful biological markers for monitoring the systemic exposure of cattle to multiple mycotoxins, as well as evaluating the effectiveness of interventions.

## 1. Introduction

Sterigmatocystin (STC) is a fungal secondary metabolite produced by fungi of the genera *Aspergillus* and *Penicillium*. STC is the end product of a biosynthetic pathway in some fungal species such as *A. versicolor* and *A. nidulans*, but is also a well-known precursor of aflatoxin B1 synthesis in various other fungal species [1,2,3]. STC has been shown to be genotoxic and potentially carcinogenic in studies with experimental animals [4] and exerts teratogenic effects at higher exposure levels [5]. The European Food Safety Authority (EFSA) recently concluded that despite its potential carcinogenic effects, STC is of minor concern to human health in Europe due to its limited prevalence in European food commodities, and subsequently there is a high margin of exposure (MoE) index. However, at the same time, the EFSA concluded that there were not sufficient data to draw conclusions about animal exposure and potential adverse health effects in animals, including cattle [4]. STC is frequently reported as a contaminant in feeds (e.g., grains, maize, and rice straw), but data on its adverse health effects in cattle are scarce, and hence maximum exposure limits have not been established.

Exposure to mycotoxins is usually assessed by analyzing feed materials of total mixed rations. An alternative approach is the measurement of toxin concentrations in biological samples (such as urine) that reflect the individual exposure levels [6,7]. Previously, we established a urine monitoring system for the mycotoxin zearalenone (ZEN) and its metabolites in cattle [8,9]. In this study, we performed liquid chromatography-tandem mass spectrometry (LC-MS/MS) of urine samples collected from these cattle herds to investigate the potential co-exposure to STC. We also used this approach to assess the efficacy of a commercial mycotoxin adsorbent (MA).

## 2. Results 

### 2.1. Urinary Metabolites of STC

To establish an analytical method for the quantification of STC in urine, preliminary trials for STC extraction were conducted, including a comparison of a direct extraction of STC and a pre-incubation of the urine samples with β-glucuronidase/arylsulfatase. The results of these analyses are presented in Table 1 and clearly indicate that the major urinary metabolite of STC is the conjugate with glucuronic acid. Therefore, in all forthcoming analyses, the urine samples were pre-incubated with β-glucuronidase/arylsulfatase before the extraction of STC.

**Table 1:** Results of the effects of the pre-incubation of urine samples with β-glucuronidase/arylsulfatase on the measurable urinary sterigmatocystin (STC) concentrations.

Urine sample	Urinary STC concentrations (pg/mg creatinine)	
	β-glucuronidase/arylsulfatase pre-incubation	
	With	Without
**Herd 1**		
1	116.2	No peak
2	95.6	No peak
3	138.3	No peak
4	49.6	No peak
5	93.5	No peak
Mean (±SEM)	98.6 ± 14.7	−
**Herd 2**		
1	46.8	No peak
2	29.1	1.6
3	63.3	No peak
4	35.4	2.6
5	35.8	No peak
Mean (±SEM)	42.1 ± 6.0	2.1 ± 0.7

### 2.2. Concentrations of STC in Urine and Feed Samples

In the current study, three groups of six cattle of the same age (23 months) and similar body weight (550–600 kg) were randomly selected from Herds 1 and 2 and divided into three treatments that differed in feed supplementation as follows: MA1, fed MA mixed with concentrate; MA2, fed MA applied as topdressing on the concentrate; and control without MA supplementation. Clinical signs of toxicity associated with mycotoxin exposure were not reported by the owners of the animals used in the trial, with the exception of chronic diarrhea in Herd 1 cattle due to unknown causes. The STC concentrations in rice straw and concentrate are shown in Table 2 together with the urinary STC concentrations. The STC concentrations in straw were two- to five-fold higher in Herd 1 than in Herd 2 at each sampling time. However, the STC concentrations in all samples of dietary concentrate from both the herds were below the detection limits of the assay (0.01 mg/kg). STC concentrations in the urine of Herd 1 control group were significantly higher (*p* < 0.05) than those in Herd 2 control group on Day 0 and the third sampling day (Day 56 in Herd 1 and Day 50 in Herd 2). In Herd 1, there were significant differences in urine STC concentrations between MA2 and the control group on Day 58 (*p* < 0.05), and between MA1 and the other two groups on Day 72 (*p* < 0.05).

### 2.3. Fungal Cultures

**Figure 1:**
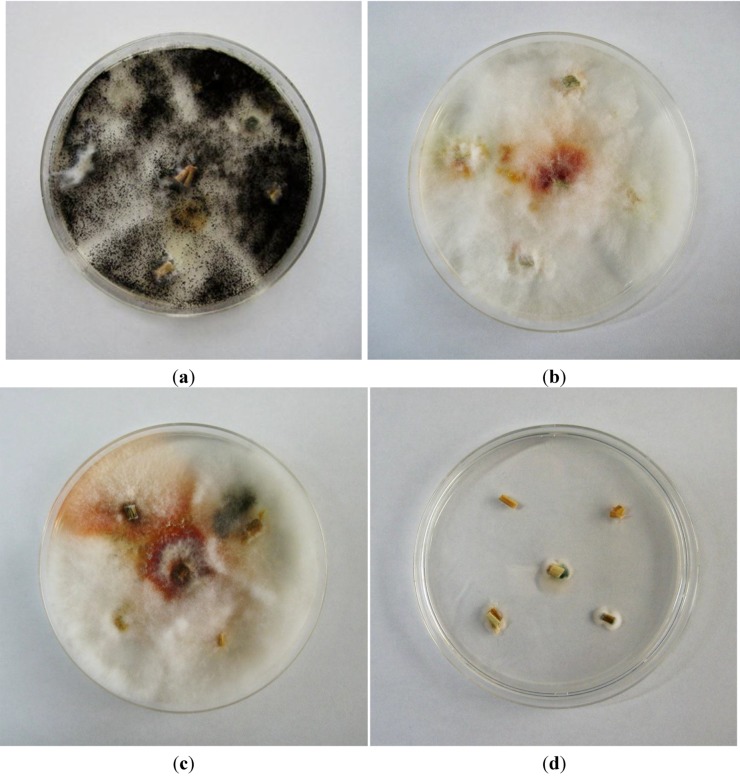
Czapek Dox agar culture showing typical colonies of *Aspergillus niger* in straw collected on Day 0 from Herd 1 (**a**); Czapek Dox agar culture showing typical colonies of *Fusarium graminearum* species complex in straw collected on Day 16 (**b**) and Day 72 (**c**) from Herd 1; Czapek Dox agar culture showing small colonies of *Penicillium spp.* in straw collected on Day 50 from Herd 2 (**d**).

The results for fungal cultures from the collected straw are shown in Figure 1. Fungal colonies from Herd 1 on Day 0 were almost exclusively *Aspergillus niger*, but also included a small colony of *Fusarium graminearum* species complex*,* which was expected, because Fusaria predominantly colonize plant material during the pre-harvest stage (Figure 1a). Fungal colonies were observed on the straw collected from Herd 1 on Days 16 (Figure 1b) and 72 (Figure 1c) and showed typical characteristics of the *F. graminearum* species complex. However, typical colonies of *Aspergillus versicolor* or *Aspergillus nidulans* were not observed on any of the straw samples from Herd 1. Fungal contamination of straw collected from Herd 2 was very low; only one very small colony of *Penicillium* sp. was observed (Figure 1d); these findings confirm the analytical results, as in the rice straw of this herd only very low amounts of STC could be detected that may have resulted from previous, no longer traceable, fungal invasion.

**Table 2:** Sterigmatocystin (STC) concentrations (measured as pg/mg creatinine) determined using liquid chromatography-tandem mass spectrometry in the urine of cattle and the effects of the application of a mycotoxin adsorbent (MA).

	Samples	Day 0	Day 16	Day 58	Day 72
Herd 1 (zearalenone (ZEN) contamination)	STC in straw; (mg/kg)	0.15	0.17	0.24	New: 0.04; Old: 0.21
	STC in concentrate; (mg/kg)	<0.01	<0.01	<0.01	NT
	STC in urine				
	MA1	327 ± 26	209 ± 22	569 ± 111	33 ± 8 ^a^
	MA2	247 ± 57	239 ± 28	416 ± 62 ^c^	258 ± 33 ^b^
	Control	190 ± 43 ^a^	182 ± 43	898 ± 97 ^a,d^	354 ± 56 ^b^
	**Samples**	**Day 0**	**Day 16**	**Day 50**	
Herd 2 (no ZEN contamination)	STC in straw (mg/kg)	0.06	0.03	<0.01	
	STC in concentrate (mg/kg)	<0.01	<0.01	<0.01	
	STC in urine				
	MA1	67 ± 22	147 ± 39	42 ± 19	
	MA2	76 ± 22	86 ± 21	0	
	Control	20 ± 13 ^b^	62 ± 23	0 ^b^	

Within rows, different superscript letters (a–b, c–d) indicate significant differences (*p* < 0.05).

## 3. Discussion

Rice straw is the most important roughage used for beef cattle production in Japan, and STC is a major mycotoxin produced in rice. The harmful or chronic effects of STC in cattle are not well understood, and the toxin is not regulated or controlled in Japan. Our objectives were to provide preliminary data on the potential contamination of rice straw with STC and to assess the systemic exposure of cattle by analyzing urine samples.

A comparison of the measurable STC concentration in rice straw confirmed the contamination with STC, albeit at rather low levels, whereas no STC could be measured in the concentrate added to the diet. The analysis of urine samples from exposed animals indicates that STC is not degraded in the rumen and reaches the liver. This is in contrast to various other mycotoxins that are successfully inactivated by the rumen microorganisms, a process which protects the animal from exposure to various feed contaminations. This is in line with the clinical observations that ruminating cattle is less sensitive to many mycotoxins, including for example ochratoxin A and the group of trichothecenes. The most prominent example for another mycotoxin that is not degraded by the rumen flora is fumonisin B1 [10]. The current study could not answer the question of what fraction of the parent STC reaches the systemic circulation. Our preliminary studies clearly indicated that STC is extensively conjugated in the liver, presumably to glucuronic acid, as only trace amounts of the free mycotoxin could be detected in urine. This finding is of clinical relevance, as pre-systemic elimination of conjugates and their excretion either by bile or with urine (as measured here), still effectively can reduce the amount of the toxin reaching the systemic circulation. The lack of significant adverse clinical symptoms in the exposed animals in this study seems to support this hypothesis.

There is a general consensus that most contaminated feed samples contain more than one mycotoxin [11,12,13]. Feed commodities might be contaminated with different types of fungi, each of which produce mycotoxins, and hence compound feeds contain many different products that contribute to the final mycotoxin profile [10,13]. For example, Warth *et al.* [14] recently reported that a commercial maize mill sample from Burkina Faso in Africa contained 6 μg/kg STC and 44 μg/kg ZEN, as well as 27 other mycotoxins. The samples investigated in this study had previously been shown to contain also the *Fusarium* toxin zearalenone, and we observed that the chronic diarrhea in cattle of Herd 1 (MA1 group) improved between Days 58 and 72, a period during which the cattle received new rice straw, less contaminated with zearalenone [9]. Therefore, although many factors might have contributed to the improvement in the physical condition of the animals, the higher level of STC in rice straw from Herd 1, possibly in combination with ZEN contamination (>8 mg/kg), might be one possible reason for the continuous diarrhea in Herd 1 cattle. Vesonder and Horn [15] previously reported acute clinical signs, including bloody diarrhea and death, in dairy cattle given STC-contaminated (8 mg/kg) feed infected with *A. versicolor*. The STC concentrations measured in our study were considerably lower, which might explain why no apparent severe clinical signs other than chronic diarrhea, which could also be attributed to other microbial infections, were noted in this study.

The growth of *Fusarium spp.* was correlated with ZEN concentrations in the straw sample from Herd 1, as we reported previously [9]. Interestingly, we did not observe the presence of *A*. *versicolor* or *A. nidulans*, both of which are known producers of STC, in the rice straw, even on Day 0. The only fungi present were *A. niger* and a small colony of *Fusarium* spp. These results indicate that fungal infection patterns differ, even within the same lot of commercially available straw, and that the contamination patterns of each mycotoxin might largely depend on the type of dominant fungus in the feed. Rank *et al.* [16] recently stated that STC is a key metabolite in mycotoxin research, and that new species that produce this toxin might be found. Therefore, although it is unclear why no fungal species known to produce STC were detected in the present study, more detailed studies that include different culture methods or molecular tools for fungal identification may be able to explain such apparent inconsistencies in the future.

Although we previously could report that the applied commercial mycotoxin adsorbent (mixed with the concentrate or given as topdressing) significantly reduced ZEN concentrations in the urine [8,9], no comparable protective effects of supplementary MA on STC absorption could be observed when measuring the STC contamination in urine as a marker. This result confirms the usefulness of urine analysis for the objective evaluation of the effects of supplemental MA on the bioavailability of mycotoxins. This finding also shows that the efficacy of MA supplementation, as a measure to prevent mycotoxin absorption, needs to be assessed for all individual mycotoxins, as significant differences can be expected.

## 4. Materials and Methods

Animals were cared for according to the Guide for the Care and Use of Laboratory Animals (Joint Faculty of Veterinary Medicine, Kagoshima University, Kagoshima, Japan).

### 4.1. Chemicals and Solvents

STC was purchased from MP Biomedicals (Heidelberg, Germany). Stock solutions of 1 μg/mL STC in acetonitrile were stored in the dark at 4 °C. Ammonium acetate and high performance liquid chromatography (HPLC)-grade methanol were purchased from Wako Pure Chemicals Industries, Ltd. (Osaka, Japan); β-glucuronidase/arylsulfatase solution was purchased from Merck (Darmstadt, Germany). Sodium acetate was purchased from Kanto Chemical Co., Inc. (Tokyo, Japan). Tris was purchased from Nakalai Tesque, Inc. (Kyoto, Japan).

### 4.2. Cattle Herds and Sample Collection

Two herds of female Japanese Black cattle kept for fattening in Kagoshima Prefecture, Japan, were included in this study, as described in more detail in our previous work [9]. Herds 1 and 2 were fed from the same lot of concentrate purchased from the same company, but they received different straw (Herd 1: purchased, Herd 2: self-produced). Three groups of six cattle of the same age (23 months) and similar body weight (550–600 kg) were randomly selected from both Herds 1 and 2 and divided into three treatments that differed in feed supplementation as follows: MA1, fed MA mixed with concentrate; MA2, fed MA applied as topdressing on concentrate; and control, no MA supplementation. The supplemented MA (Mycofix®Plus3.E, Biomin) was a commercially available product comprising minerals and biological constituents, including enzymes, yeast cell wall, clay, and plant extracts [17]. The maximum daily dose of MA recommended by the supplier (50 g) was provided in two 25-g doses per day for a 16-day period.

**Figure 2:**
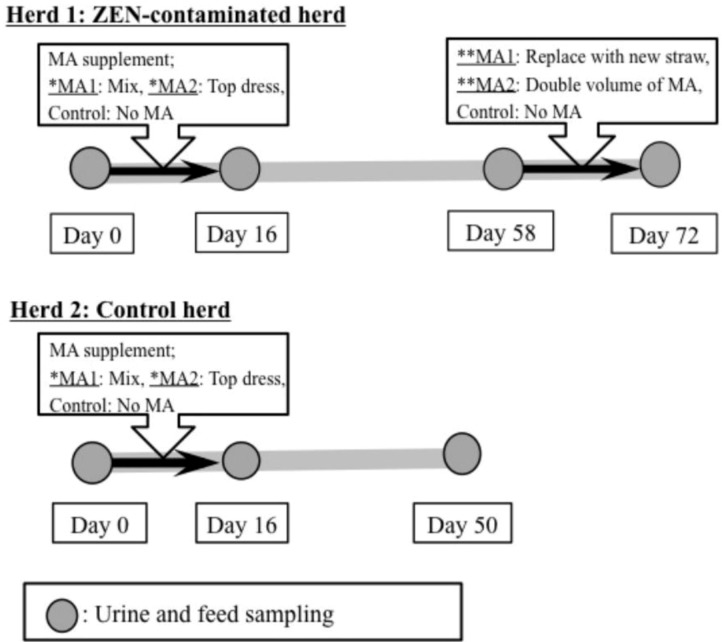
Protocol for the supplementation of dietary feed with mycotoxin adsorbent (MA) and for the sampling of urine and feed samples. Sterigmatocystin concentrations in the urine and feed samples collected at each sampling point were analyzed using liquid chromatography-tandem mass spectrometry. Herd 1: Zearalenone (ZEN)-contaminated herd that had shown persistently higher mean ZEN concentrations in the urine. Herd 2: No ZEN contamination. Both herds were fed from the same lot of concentrate, but they received different rice straw. *MA1: MA was mixed with dietary concentrate. *MA2: MA was topdressed on the dietary concentrate. **MA1: Typical straw was replaced by a new lot of straw with less contamination. **MA2: Twice the recommended volume of MA was topdressed on the dietary concentrate. Control: No MA was provided during the experimental period.

At 2 h after the morning feeding, spontaneous urine samples were collected from the animals by massaging the perineum. This activity was performed at the start of feed supplementation (Day 0), on the final day of supplementation (Day 16), and on the final day of the observation period (Day 58 for Herd 1; Day 50 for Herd 2). In addition, samples of rice straw and feed concentrate (approximately 1 kg each) were obtained from both the herds to measure STC concentrations in the feed. The protocol for MA supplementation and all sampling procedures are summarized in Figure 2. All samples were immediately placed in a cooler containing ice for protection from light and were transported to the laboratory. Urine samples were centrifuged at 500 ×*g* for 10 min at room temperature to remove the debris. The urine and feed samples were frozen at −30 °C until the analysis of STC and creatinine.

### 4.3. Analysis of STC in the Feed

STC concentrations were measured in straw and concentrate by using an API 3200 LC-MS/MS (Applied Biosystems, Tokyo, Japan) equipped with an electrospray ionization (ESI) interface and a Prominence HPLC system (Shimadzu Corp., Kyoto, Japan), according to the Food and Agricultural Materials Inspection Center (FAMIC) [18] at Shokukanken Inc., Gunnma, Japan. In brief, representative samples of stored straw (2 g) and concentrate (10 g) were homogenized and chopped into small pieces. Each sample was placed in a sample tube, to which 20 mL of 84% acetonitrile was added. The tubes were shaken for 1 h and centrifuged for 10 min at 500× *g* at room temperature. The supernatant (10 mL) was loaded onto a MultiSep 226 Aflazon+ Multifunctional column (Romer Labs, Union, MO, USA). Subsequently, 1 mL of the eluent was mixed with 1 mL acetic acid (1 + 100) and centrifuged for 5 min at 500× *g*.

Next, 10 μL supernatant was injected into the LC-MS/MS system under the following conditions: column, Gemini C18 (2 mm × 50 mm, 3 μm); oven temperature, 40 °C; eluent flow, 200 μL/min; and solvent, 0.1% aqueous formic acid (A) + 0.1% formic acid in methanol (B). An ESI probe was used in the positive mode for STC analysis. The ESI conditions were as follows: curtain gas, 20 psi; ion-spray voltage, 4500 V; turbo temperature, 600 °C; collision energy, 49.0 eV; declustering potential, 55.0 V; and entrance potential, 10.0 V. The detection limit for each analyte was 0.01 mg/kg. The mean STC recovery rates were 90.5%–93.5%.

### 4.4. Analysis of STC and Creatinine in the Urine

Concentrations of STC were determined using LC-MS/MS by using an API 2000 system (Applied Biosystems, Foster City, CA, USA) equipped with an electrospray ionization (ESI) interface and a HPLC system (1200 Series; Agilent Technologies, Santa Clara, CA, USA). In brief, the urine samples (0.5 mL) were mixed with 3.0 mL of 50 mM ammonium acetate buffer (pH 4.8) and 10 μL of glucuronidase/arylsulfatase solution and incubated for 12 h at 37 °C. After incubation, the solution was loaded onto a C18 solid-phase extraction (SPE) column (Strata, Phenomenex, Torrance, CA, USA) that had been preconditioned with 3 mL 100% MeOH and 2 mL Tris buffer. An additional 2 mL Tris buffer and 3 mL 40% MeOH were added. After elution with approximately 1 mL 80% MeOH, the volume was adjusted to exactly 1 mL.

**Figure 3:**
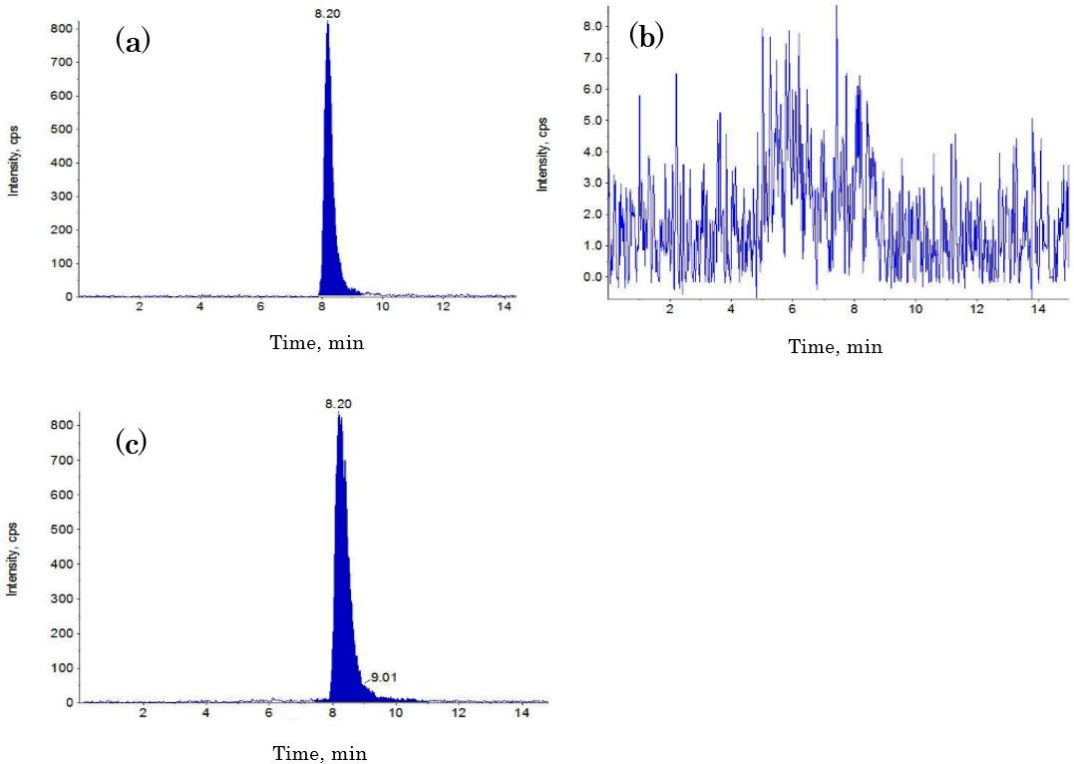
Representative liquid chromatography-tandem mass spectrometry chromatograms for (**a**) the STC standard (10 ng/mL); (**b**) no detectable level of STC in the urine sample; and (**c**) a clear peak of STC contamination in the urine sample.

Next, 20 μL of solution was injected into the LC-MS/MS system. Chromatographic separation was performed on an Inertsil ODS-3 column (4.6 i.d. × 100 mm, 5 μm; GL Sciences, Tokyo, Japan) at 40 °C. The mobile phase, which consisted of methanol/water/acetic acid (97:3:0.01, *v*/*v*/*v*), was applied (200 μL/min) to separate the analyte in the isocratic mode. The measurement time was 15 min. A multiple reaction monitoring system was used to switch STC (*m*/*z* 325.0–281.0) to the positive ion mode. Instrumental parameters were optimized for STC measurement by analyzing the corresponding standard solution at a flow rate of 10 μL/min by using a syringe pump integrated into the API-2000 MS. The electrospray conditions were as follows: curtain gas, 40 psi; ion-spray voltage, 5500 V; turbo temperature, 500 °C; collision energy, 16.0 eV; declustering potential, 6.0 V; focusing potential, 360 V; and entrance potential, 10.5 V. Nitrogen was used as a nebulizer, curtain, and collision gas. The mean recovery rate was between 85% and 120% in our assay. Representative chromatograms from the LC-MS/MS assay are shown in Figure 3.

Concentrations of creatinine in the urine were determined using a commercial kit (Sikarikit-S CRE; Kanto Chemical, Tokyo, Japan) according to the manufacturer’s instructions and measured using a clinical autoanalyzer (7700 Clinical Analyzer; Hitachi High-Tech, Tokyo, Japan). STC concentrations in the urine were expressed as a ratio to creatinine (pg STC/mg creatinine), as described previously [19,20].

### 4.5. Fungal Cultures

Fungal samples were cultured directly from the samples of infected rice straw selected from the two herds. Each straw sample (5-mm^2^ fragment) was soaked in 1% sodium hypochlorite for 1 min, rinsed in sterile water three times, plated on Czapek Dox agar (0.3% NaNO_3_, 0.1% K_2_HPO_4_, 0.05% KCl, 0.05% MgSO_4_·7H_2_O, 0.001% FeSO_4_, 3% sucrose, *w*/*v*), and incubated at 25 °C in the dark for 7 days. Fungal colonies that emerged on the straw were observed, and the fungi were identified macroscopically.

### 4.6. Statistical Analysis

Concentrations of STC in the urine collected from each herd during the three sampling events were compared among the three groups (MA1, MA2, control) by using one-way analysis of variance (ANOVA) followed by a post-hoc test performed the StatView program (Abac Concepts, Inc., Berkeley, CA, USA) to evaluate the effects of MA supplementation. The significance threshold was set at *p* < 0.05. Results for STC concentrations are expressed as means ± SE.

## 5. Conclusions

In conclusion, to our knowledge, this is the first study showing that STC present in the diet of ruminating cattle is absorbed from the intestinal tract and passes the rumen. Results also show that the measurement of urinary concentrations of mycotoxins, including STC, is a reliable tool to monitor the exposure of cattle to contaminated feed. In addition, our results indicate that MA used in this study could not sequester STC. The observed lack of benefit from MA supplementation contrasts with our previous finding of the protective effect of MA against ZEN adsorption. Considering the systemic bioavailability of STC in ruminating cattle, further field studies are required to improve our understanding of STC contamination and co-contamination with other mycotoxins and to assess the potential risk for cattle health.
